# Shortening Epitopes to Survive: The Case of SARS-CoV-2 Lambda Variant

**DOI:** 10.3390/biom11101494

**Published:** 2021-10-10

**Authors:** Stefano Pascarella, Massimo Ciccozzi, Martina Bianchi, Domenico Benvenuto, Marta Giovanetti, Roberto Cauda, Antonio Cassone

**Affiliations:** 1 Department of Biochemical Sciences “A. Rossi Fanelli”, Sapienza Università di Roma, 00185 Rome, Italy; martina.bianchi@uniroma1.it; 2 Medical Statistic and Molecular Epidemiology Unit, University of Biomedical Campus, 00128 Rome, Italy; domenicobenvenuto95@gmail.com; 3 Flavivirus Laboratory, Oswaldo Cruz Institute, Oswaldo Cruz Foundation, Rio de Janeiro 21040-360, Brazil; giovanetti.marta@gmail.com; 4 Dipartimento di Sicurezza e Bioetica, Sezione Malattie Infettive, Università Cattolica S. Cuore, 00168 Rome, Italy; roberto.cauda@unicatt.it; 5 Center of Genomics, Genetics and Biology, 53100 Siena, Italy; antonio.cassone2@gmail.com

**Keywords:** SARS-CoV-2, Lambda variant, epitope loop shortening, N-glycosylation site, interaction energy, immunoevasion

## Abstract

Among the more recently identified SARS-CoV-2 Variants of Interest (VOI) is the Lambda variant, which emerged in Peru and has rapidly spread to South American regions and the US. This variant remains poorly investigated, particularly regarding the effects of mutations on the thermodynamic parameters affecting the stability of the Spike protein and its Receptor Binding Domain. We report here an in silico study on the potential impact of the Spike protein mutations on the immuno-escape ability of the Lambda variant. Bioinformatics analysis suggests that a combination of shortening the immunogenic epitope loops and the generation of potential N-glycosylation sites may be a viable adaptation strategy, potentially allowing this emerging viral variant to escape from host immunity.

## 1. Introduction

COVID-19 is a multi-organ, systemic disease caused by a new coronavirus, SARS-CoV-2, which emerged in China, probably in the city of Wuhan, in late 2019 and has since spread worldwide to cause the first viral pandemic of the 21st century. By 31 August 2021, the cumulative number of COVID-19 cases had reached 216 million with a death toll approaching 4.5 million people (www.who.int, accessed on 3 September 2021). Through the unprecedented outflow of technological, monetary, and human resources, some efficacious and safe anti-SARS-CoV-2 vaccines have been rapidly developed (in less than one year) and proven to be safe and effective in clinical trials and real-world use [1]. Nonetheless, their effectiveness in combating this disease is being threatened by the emergence of viral variants bearing mutations in their RNA genome capable of enhancing virus transmission and escaping the immunity conferred by vaccination or acquired naturally during illness [2,3].

The most important of these variants are termed variants of interest (VOI) or concern (VOC) and are intensely investigated to elucidate the mechanisms underlying their transmission and/or resistance to neutralization by immune sera and monoclonal antibodies [4]. These variants differ from the original Wuhan reference virus in a number of mutations, of which those of the gene encoding the Spike (S) protein are of special virological and medical relevance. The S protein does indeed bind the human Angiotensin Converting Enzyme 2 (ACE2) receptor and allows the virus entry into host cells and causes infection [5]. Consequently, the antibodies which recognize the antigenic determinants (epitopes) of the S protein can neutralize the SARS-CoV-2 infection and protect from disease. The presence of such “neutralizer epitopes” plays a major role in the natural history of the SARS-CoV-2 infection; hence, their mutations are of particular concern because they could modify, reduce, or even abolish neutralization by antibodies, thus inducing immuno-escape from naturally acquired or vaccine-induced protection [6,7].

VOC and VOI are currently identified by Greek alphabet letters and/or an alfa-numerical code starting from the VOC Alfa (B.1.1.7), which bears the Spike mutation N501Y and first emerged in the UK [8]. One of the most studied is the VOC Delta (B.1.617), which emerged in India and has since spread worldwide to become the now dominant SARS-CoV-2 strain in most countries [9].

Among the more recently identified VOI, the Lambda variant (C.37) emerged in Peru, rapidly spread to South America and is now present in several other countries, including the US [10]. This new variant warrants attention for its potential epidemic impact but remains poorly investigated, particularly regarding the effects of mutations on the thermodynamic parameters affecting the stability of the Spike protein and its Receptor Binding Domain (RBD). Variations in these parameters, if consistent, have been shown to predict relevant changes in SARS-CoV-2 transmission and contagiousness [11,12]. In addition, a sound, comparative bioinformatic approach can be predictive of the possibility that mutations of the neutralizer epitopes bring substantial immuno-escape by the variant. Following this rationale, we have here compared the Spike sequences of the Delta and Lambda variants and analyzed their mutations from the reference Wuhan strain in an effort to predict the mutation impact on the variant Spike stability, transmission, and antibody immuno-escape ability. Although theoretical, our results can inform appropriate experimental and clinical confirmatory investigations.

## 2. Materials and Methods

The Spike sequence representative of the Lambda and Delta variants was retrieved from the GISAID [13] translated protein set of the isolate identified by the codes EPI_ISL_1629764 and EPI_ISL_1634920, respectively. The reference Wuhan Spike sequence is labelled by the RefSeq [14] code yp_009724390 corresponding to the UniProt [15] code P0TDC2. The Lambda Spike structure was modelled using the method available in the web server Swiss-Model [16] using as a starting structure the coordinate set identified by the PDB code 7KRS. This structure was selected by Swiss-Model as the best trimeric template. This coordinate set contains the Cryo-EM structure of the Spike mutant D614G. The model was built using the sequence mode: the target sequence was given as an input and the program searches for the best templates, from which the user selects the one to be used for model building. At the end of the calculations, the stereochemistry and residue contacts in the model are automatically optimized. 

Energy minimization of the model complexes between the Spike N-terminal domain (NTD) and the antibody was applied to remove possible residue steric overlaps at the interface. The energy minimization protocol embedded in the molecular graphics program Swiss-PdbViewer [17] was applied. The protocol relies on the GROMOS96 43B1 force field, cutoff 10 Å, and 100 steps of the steepest descent minimization, followed by 1000 steps of the conjugate gradients in vacuo. The minimization was stopped if the energy difference between two consecutive steps was lower than 0.05 kJ/mol. Only residues at the interface were minimized. This forcefield does not include the parameters to describe glycans, which were therefore ignored during minimization. However, the glycans included in the model do not interfere with the interactions at the interface of the complex.

The server DynaMut [18] was utilized to predict the impact of point mutations on the stability of the Spike structure. DynaMut relies on the Normal Mode Analysis of the molecular dynamics of the protein to assess the stability variation upon mutation. Changes in stability are measured as ΔΔG (kcal/mol); positive and negative values indicate stabilization and destabilization, respectively. The server does not require any parameter input from the user. 

GlycoPred [19] and NetNGlyc [20] were used to predict potential glycosylation sites. GlycoPred utilizes a Random Forest predictor which is reported to reach 92.8% of correct predictions and a Mathews Correlation Cofficient equal to 0.85 for the N-glycosilation sites. NetNGlyc is based on artificial neural networks. Predictions were made using the recommended threshold score of 0.5. 

Protein-protein interaction energy was predicted with the method implemented in PRODIGY [21] using the default parameters (temperature 25 °C). This method predicts the binding energy and affinity of a protein complex on the base of the number and type of interfacial residue-residue interactions. 

Structural analysis and visualization were carried out with PyMOL [22] or UCSF Chimera [23].

Discontinuous B-cell epitopes were predicted with the methods implemented in the software DiscoTope [24] and BePro [25]. DiscoTope predicts potential B-cell epitopes by attribution of an epitope propensity score to each residue and by analysis of the corresponding spatial neighborhood along with surface exposure. BePro utilizes a similar strategy. DiscoTope predictions were carried out with the default threshold for the combined score of −3.7, which corresponds to an expected sensitivity and specificity equal to 0.47 and 0.75, respectively. BePro epitope assignment was carried out using a score threshold equal to 0.95. For reference, it has been reported that a threshold equal to 1.3 corresponds to a sensitivity >0.3 and a specificity >0.9.

Computational alanine scanning of the residues at the interfaces between the Spike NTD and the antibody was obtained through the webserver DrugScore^PPI^ [26]. The method available in the server provides a fast and accurate system to predict the binding free energy changes upon alanine mutations at protein-protein interfaces using a knowledge-based scoring function. The method does not require any parameter input from the user. 

## 3. Results

**Figure 1:**
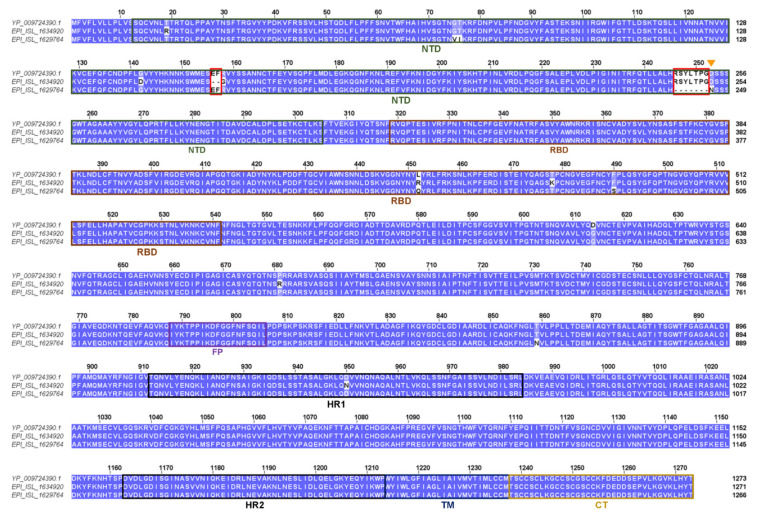
Sequence comparison between the Wuhan reference (yp_009724390.1) and the Lambda (EPI_ISL_1629764) and Delta (EPI_ISL_1634920) variant S proteins. Red boxes indicate the deletions within the N-terminal domains (NTD) while the orange triangle marks the potential new N-glycosylation site in the Lambda variant. Colored, labelled boxes indicate the sequence position of the Spike domains. NTD, RBD, FP, HR1, HR2, TM, and CT mean N-terminal domain, Receptor Binding Domain, Fusion peptides, Heptad repeat 1 and 2, Transmembrane domain, and C-terminal domain, respectively. Conserved columns have a blue background.

A homology model for the Lambda variant S protein was built as described in Materials and Methods. Analysis of the mutation structural context and the potential functional impact was carried out. Figure 1 reports the sequence alignment between the Lambda and the Delta, and the reference S proteins showing their different sets of mutations. As the thermodynamic parameters of the Delta variant mutations were previously reported [27], we have focused here on the Lambda variant.

The Lambda mutations G75V and T76I occur at an exposed loop connecting two short antiparallel β-strands (Table 1). The effect of each of the two mutations is predicted to be stabilizing. This loop is in contact with the loop encompassed by the sequence positions 246–280 which is one of the epitopes recognized by mAbs [28]. Interestingly, the deletion 246–252 (corresponding to the sequence RSYLTPG) occurs within this loop. To predict the potential impact of the deletion onto the NTD affinity to a human mAbs, the complex between the SARS-CoV-2 Spike and the 4A8 Ab deposited as 7C2L in PDB was used as a case study. 

**Figure 2:**
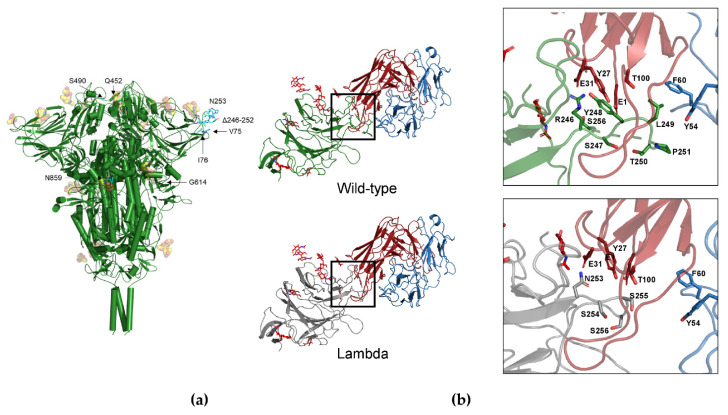
(**a**) Map of the Lambda mutations of the trimeric Spike structure. Only one mutation per monomer is indicated. Glycans are indicated as orange sphere models (**b**) interface chain A NTD-4A8. The mutant NTD was modelled via Swiss-Model and superposed to the wild-type domain of the complex. Interface interactions and energies calculated by PRODIGY were compared. Comparison of the interfaces between the wild-type (green cartoon, top) and the Lambda NTD (grey cartoon, bottom) and the mAb 48A at the deletion region. Red and blue cartoons indicate the heavy and light chain, respectively. Side chains in the deleted loop and the interacting residues are indicated with stick models and labelled. Sequence numbering refers to the wild-type Spike. Glycans are reported as red sticks.

The mutant NTD was modelled via Swiss-Model and superposed to the wild-type domain of the complex. Within the Spike trimer, the NTD model was built using the chain A of 7C2L as the template. To remove possible steric overlaps of the interfacial residues, the Swiss-PdbViewer energy minimization was applied. The interface interactions and energies calculated by PRODIGY were compared (Table 2 and Figure 2). Overall, the partial deletion of the loop is predicted to weaken interaction with the 4A8 antibody with a consequent decrease in binding affinity owing to the loss of several interactions. In particular, the deletion in Lambda NTD removes interactions that in the wild-type complex take place between L249 and F60, Y54 of the 4A8 light chain. Moreover, a salt bridge and a π-cation interaction between R246 and 4A8 E31 and Y27, respectively, disappear in the Lambda variant (Figure 2).

**Figure 3:**
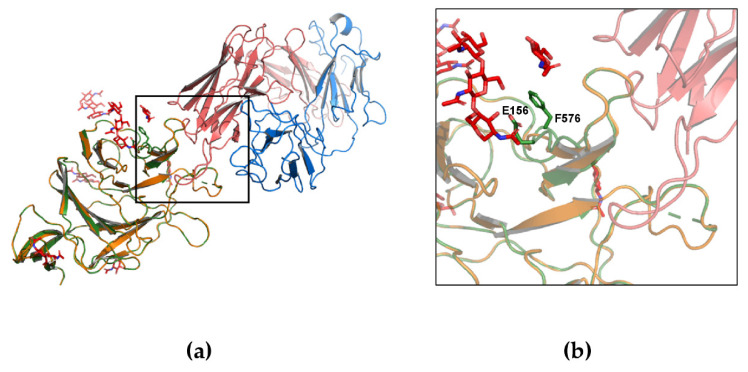
Comparison between the reference (chain A) and model Delta NTDs in complex with 4A8. (**a**) Cartoon model of the complex: reference and Delta NTDs are represented as green and orange cartoon models, respectively. A8 heavy and light chain are depicted with red and blue cartoons, respectively. (**b**) Detail of the NTD-4A8 antibody interface. The reference Spike residues that are deleted in the Delta variant are indicated as stick models and labelled. Glycans are displayed as red sticks models.

Interestingly, the Delta Spike also displays a two-residue deletion in the NTD at positions 156–157. This deletion shortens the β-strand 154–160. For comparison with the Lambda variant, the complex NTD-4A8 was also modelled for the Delta Spike (Figure 3) and the interaction energy calculated. Additionally, in this case chain A of the 7C2L trimer was used as a template. Apparently, the predicted affinity for this specific antibody does not seem to change significantly in this variant (Table 2). Overall, the two-residue deletion in the Delta variant does not seem to significantly alter the NTD conformation, although it occurs outside the loop 141–154 that takes part in the interaction with 4A8. Although glycans were included in the models (Figure 2 and Figure 3), the methods applied take into account only standard residues. However, the glycan molecules present in the template and in the model do not appear to interfere with the interactions taking place at the interface of the RBD-Ab complex.

This observation has been corroborated by the DrugScore^PPI^ in silico alanine scanning. The contribution of the S residues at the interface between the NTD and the 4A8 antibody to the overall binding energy was predicted as ΔΔG upon the mutation of each residue into alanine. The results are reported in Table S1. Interestingly, R246 and Y248, belonging to the deleted loop in the Lambda variant, are predicted to contribute significantly to the binding energy. Indeed, R246 forms a salt bridge with Glu31 of the 4A8 heavy chain (Figure 2b). In the Lambda variant, these stabilizing effects disappear because of the deletion of the key residues. Interestingly, the deletion of R158 in the Delta variant is predicted as not significantly weakening the interaction with 4A8 as this residue is attributed to a small ΔΔG (Table S1). 

To test the possible impact of the NTD deletion on the S protein antigenicity, the potential B-cell epitopes predicted for the reference and Lambda Spike were compared. DiscoTope and BePro were applied to the 3-dimensional structures of the two proteins. Both methods predict the presence of a B-cell neutralizer epitope in the sequence of the reference S protein at the positions corresponding to the deleted loop in the Lambda S protein (Figure 2b and Table S2). This result is in agreement with the observations derived from the analysis of the Spike-4A8 complex and suggests that the loss of a neutralizer epitope decreases the immunogenic potential of the Lambda S protein. On the contrary, the Delta variant displays a potential epitope pattern in the NTD similar to that predicted for the reference S protein (Table S2).

In contrast to the above, the mutations in the RBD region of the Lambda variant (L452Q and F490S) are predicted to have little impact on the domain thermodynamic stability (Table 1). Both replacements change a hydrophobic residue into a hydrophilic one, inducing a local increase in the surface hydrophilicity. The sites are exposed to the solvent close to the interface RBD-ACE2. The binding affinity of the mutant and wild-type RBD is almost identical according to the PRODIGY predictions (Table S3). Finally, of the two mutations within the S1 and S2 subunits, i.e., D614G and T859N, the former was extensively described and attributed with transmissibility increase [11,12] while the latter occurs in the S2 subunit part of the Spike in a short β-strand in a position that is about 8 Å distant from the G614 of the adjacent S1 subunit. The impact of the mutation on the thermodynamic stability is predicted to be neglectable (Table 1). However, this mutation introduces a H-bond to K847 and a long-distance effect on the S properties cannot be excluded. Indeed, the allosteric effects of the mutations in this portion of the S protein have been suggested and studied [29,30].

Finally, it should be noted that the Lambda mutation D253N is predicted as a potential N-glycosylation site by GlycoPred and NetNGlyc while neither Lambda T859N nor Delta D950N are. 

## 4. Discussion

Overall, our in silico analysis suggests that the point mutations characterizing the Lambda variant do not seem to directly influence the RBD affinity for the ACE2 receptor, thus making uncertain the relevant impact of these mutations on virus transmission, unless the mutations in the S2 domain have a long-distance effect on the ACE2 affinity. This suggestion is in substantial agreement with the little differences in the experimentally determined values of the binding affinity of S protein to the ACE2 receptor of the Delta and Lambda variants recently reported by Liu and collaborators [31]. 

The most evident and likely functionally impacting the change of the Lambda variant is represented by the 246–252 deletion. This amino acid loss could confer to the virus an enhancing capacity to escape from the host immune response by two theoretical, though likely and already reported, strategies: (i) shortening or fully deleting neutralizer epitopes located in the loops; (ii) exploiting increased glycosylation. Variations in the Spike protein epitopes and glycosylation profile during the virus transmission have been already described [32]. Of interest is also the recent report that the convalescent sera from recipients of the Pfizer-BioNTech vaccine lose reactivity towards the RBD of the Lambda variant while substantially retaining it towards the RBD of the Delta variant [31]. This observation is in keeping with the suggestion, based on the vitro-immunological data [10] that the Lambda resistance to the vaccine-induced neutralization is indeed determined by the 246–252 deletion in the NTD of the S protein. Overall, the role of the neutralizer epitopes in the NTD S domain appears to be a relevant one in the protection, contrasting previous considerations about the dominance of RBD mutations in this critical aspect of the current pandemic [32,33]. 

We recognize that the scientific content of this paper, which reports results of in silico analysis, is limited by the absence of experimental and/or clinical data. However, there is a rather large agreement of our in silico data with the experimental virological and immunological findings published by others, as detailed in the quoted manuscripts. Such concordance, though quite indirect, somewhat strengthens the predictive value of suitably collected and deployed bioinformatic approaches to inform about the role of the SARS-CoV-2 VOC in epidemic transmission and control by vaccines.

## Figures and Tables

**Table 1:** Summary of analysis of Spike protein mutations by DynaMut. The differences in free energy were estimated in comparison to the Wuhan sequence. The program does not include deletion mutants.

Mutation	Structural Position	∆∆G (kcal/mol)	Local Interactions
G75V	NTD. β-hairpin. Exposed	0.214 (moderately stabilizing)	Hydrophobic interaction to V57
T76I	NTD. β-hairpin. Buried	1.248 (highly stabilizing)	Hydrophobic interaction to L244, W251. Adjacent to the deleted loop 246–252
Δ246–252	NTD. Loop	Not applicable	Deleted loop involved in interaction with human antibodies (4A8, FC05)
D253N	NTD deletion C-terminal side	0.07 (neutral)	Introduces a potential N-glycosylation site
L452Q	Receptor binding motif	0.295 (moderately stabilizing)	Increases surface hydrophilicity. Close to the interface to ACE2
F490S	Receptor binding motif	−0.039 (neutral)	Increases surface hydrophilicity. Possible polar interaction to E484. Close to the interface to ACE2
D614G	SD2	Not tested	Extensively characterized. Influences conformational flexibility and susceptibility to proteolytic activation.
T859N	S2. Short β-strand	−0.037 (neutral)	H-bond to K847 of the same chain. In proximity of G614 of the other chain

**Table 2:** PRODIGY prediction of the interaction energies in the complexes between wild-type reference and Lambda Spike NTD with 4A8 Ab.

NTD	∆G (kcal/mol)	K_d_ (M) at 25 °C	No of Interface Contacts ^a^
Wild type	−11.3	5.6 10^−9^	68
Lambda	−10.6	1.8 10^−8^	56
Delta	−11.7	4.0 10^−9^	69

^a^ Overall number of contacts between residues from NTD and 48A. These contacts include different types of interactions, such as electrostatic, van der Waals, polar, and the like.

## Data Availability

Publicly available datasets were analyzed in this study. Data presented in this study are available on request from the corresponding authors.

## Supplementary Materials

The following are available online at https://www.mdpi.com/article/10.3390/biom11101494/s1, Table S1: Variation of binding energy of the residues at the interface between NTD and 4A8 calculated by DrugScore^PPI^ alanine scanning; Table S2: Comparison of the B-cell epitope predictions in the NTD; Table S3: PRODIGY prediction of interaction energy between ACE2 and Spike RBD of Reference, Lambda, and Delta variants
