# Supplementary material for: Shortening Epitopes to Survive: The Case of SARS-CoV-2 Lambda Variant
Source: Biomolecules. 2021 Oct 10;11(10):1494. doi: 10.3390/biom11101494 (PMC8533401; doi:10.3390/biom11101494)
Supplement: Supplementary file 1 [file biomolecules-11-01494-s001.zip › biomolecules-1388132-supplementary.pdf]

Table S1. Variation of binding energy of the residues at the interface between NTD and 4A8 calculated by DrugScore^{PPI} alanine scanning.

	**Reference**	**Lambda**	**Delta**
Interface residues	ΔΔG (kcal/mol)	ΔΔG (kcal/mol)	ΔΔG (kcal/mol)
V143	0.18	0.19	0.18
Y144	0.30	0.37	0.31
Y145	1.64	1.73	1.70
H146	0.06	0.07	0.01
K147	1.67	1.64	1.67
N148	0.33	0.33	0.33
K150	0.60	0.72	0.72
S151	0.13	0.13	0.21
W152	1.73	1.72	1.57
R158	0.09	0.07	Δ
H245	0.13	0.06	0.16
R246	1.20	Δ	1.81
S247	0.15	Δ	0.13
Y248	2.96	Δ	3.06
L249	1.16	1.16	1.09
T250	0.16	0.16	0.13
S256	0.09	0.09	0.09

Table S2. Epitope prediction. Sequence position of the discontinuous epitopes predicted for the wild type, the Lambda and Delta variant NTDs.

**Reference**[a]		**Lambda**[a]		**Delta**[a]	
DiscoTope	BePro	DiscoTope	BePro	DiscoTope	BePro
X	23-25	X	23-25	X	22-26
71-74	71-73	72-74	72-73	71-75	71-73
146-151	146-153	146-151	146-153	146-151	146-152
X	173-176	X	173-176	X	171-174
181-185	182-187	182-184	182-187	180-184	180-185
X	209-213	X	211-213	X	207-211
247-252	248-252	X	X	247-252	246-252

[a] X means not predicted as epitope

Table S3. PRODIGY prediction of interaction energy between ACE2 and Spike Receptor Binding Domain (RBD) of Reference, Lambda and Delta variants

**RBD**	**ΔG (kcal/mol)**	**K_d (M) at 25°C**	**No of interface contacts[a]**
Reference	-11.9	1.9 10^{-9}	66
Lambda	-12.1	1.4 10^{-9}	66
Delta	-11.9	1.9 10^{-9}	66

[a] Overall number of contacts between residues from RBD and ACE2. These contacts include different type of interactions such as electrostatic, van der Walls, polar, and the like.
